# Increased Red Cell Superoxide Dismutase Activity Is Associated with Cancer Risk: A Hidaka Cohort Study

**DOI:** 10.1158/2767-9764.CRC-24-0301

**Published:** 2024-11-04

**Authors:** Shin-ichiro Tanaka, Yoshio Fujioka, Takeshi Tsujino, Tatsuro Ishida, Ken-ichi Hirata

**Affiliations:** 1 Department of Internal Medicine, Toyooka Hospital Hidaka Clinic, Hyogo, Japan.; 2 Division of Clinical Nutrition, Faculty of Nutrition, Kobe Gakuin University, Kobe, Japan.; 3 Department of Pharmacy, School of Pharmacy, Hyogo Medical University, Kobe, Japan.; 4 Division of Nursing Practice, Kobe University Graduate School of Health Sciences, Kobe, Japan.; 5 Division of Cardiovascular Medicine, Kobe University Graduate School of Medicine, Kobe, Japan.

## Abstract

Superoxide dismutase (SOD) catalyzes the highly reactive superoxide anion to form hydrogen peroxide, which facilitates cell proliferation and death. We investigated whether red cell SOD (R-SOD) activity is associated with an increased risk of cancer in a Japanese general population. We prospectively analyzed data from 1,921 participants (800 men and 1,121 women; age, 58.7 ± 14.7 years) in a Hidaka cohort study. After a median follow-up period of 10.9 years, 160 participants had developed cancer. The Cox proportional hazards model was used to estimate quartile-specific HRs and 95% confidential intervals (CI) for cancer risk. After adjustment for potential cancer risk factors including age, sex, current smoking habit, alcohol use, physical activities, body mass index, plasma immunoreactive insulin, and non–high-density lipoprotein cholesterol levels, we found a significant association between R-SOD activity and an increased risk of cancer (HR, 1.61; 95% CI, 1.03–2.52; *P* = 0.037). In analyses conducted separately by sex, a significant association was found in men (HR, 2.49; 95% CI, 1.35–4.59; *P* = 0.003) but not women (HR, 1.46; 95% CI, 0.70–3.05; *P* = 0.320). After excluding participants who developed cancer within 5 years of the baseline survey, the association was more evident in men (HR, 4.64; 95% CI, 1.88–11.45; *P* = 0.001). We found no association with cancer risk in women (HR, 1.01; 95% CI, 0.39–2.65; *P* = 0.983). Increased R-SOD activities were associated with an increased risk of cancer, particularly in men in this population.

**Significance:**

Our study is the first to show that increased R-SOD activity is associated with a significantly higher cancer risk in men but not in women. Antioxidative enzymes such as SOD are essential for maintaining cellular redox balance. Their roles in cancer development and prevention are yet to be fully elucidated.

## Introduction

Reactive oxygen species (ROS), including the superoxide anion, hydrogen peroxide, and hydroxyl radicals, have been implicated in the pathogenesis of cardiovascular disease (1–3), cancer (4–6), neurodegenerative disease (7, 8), and a variety of other conditions including infectious diseases (9, 10). ROS also act as signal transduction molecules in cells and play important roles in cell proliferation (11, 12) and death (13). Therefore, ROS concentrations determine the survival or death of cancer cells (14).

Superoxide dismutase (SOD), first identified by McCord and Fridovich in bovine erythrocytes (15), dismutates superoxide anion to form hydrogen peroxide. The hydrogen peroxide, in the presence of ferrous ions, is subsequently converted to highly reactive hydroxyl radicals, which damage DNA (16). Alternatively, it is converted to water through the actions of the antioxidant enzymes catalase and glutathione peroxidase (17). Hydrogen peroxide also acts as a signal transduction molecule, and SOD has been found to be essential for its mediating role in growth factor signaling and cell proliferation (18).

In humans, SOD has three isoforms: copper/zinc SOD, manganese SOD, and extracellular SOD (19). Copper/zinc SOD is constitutively expressed in the cytosol and accounts for the majority of SOD activities in cells (19, 20). Measurements of the activities of cytosolic SOD such as red cell SOD (R-SOD) may indicate the general antioxidative status of individuals.

The activities of R-SOD and other red cell antioxidative enzymes have been measured to investigate relationships between ROS and various conditions including cardiovascular disease (21), neurodegenerative disease (8), and cancer (22). However, the role of SOD in cancer progression remains controversial, and basic and clinical studies have yielded inconsistent findings (18, 23–26). Furthermore, only limited information is available about SOD and cancer development in the general population (27). Therefore, in the present study, we tested the hypothesis that baseline R-SOD activity is associated with a future risk of cancer, by analyzing 10.9 years of follow-up data for community-dwelling individuals.

## Materials and Methods

### Study population

**Figure 1:**
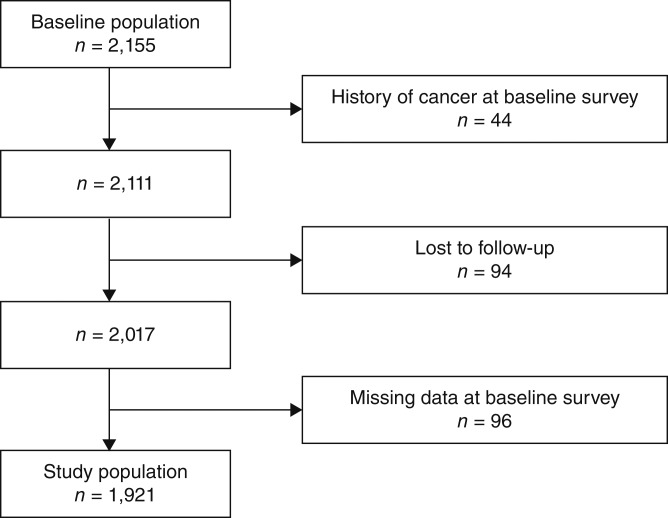
Study population.

The baseline survey was conducted in 1993 as a cardiovascular disease survey in Hidaka town, a rural community in Japan (3). A total of 2,155 individuals ages ≥20 years were enrolled in this survey. Of these, 44 had a history of cancer, 94 were lost to follow-up, and 96 had missing data, making them ineligible for the present study; therefore, data from the remaining 1,921 participants were included in our analysis (Fig. 1).

### Ethics statement

The study was conducted in accordance with the Helsinki Declaration and approved by the institutional ethical review board of Hidaka Medical Center (currently the Hidaka Clinic). Written informed consent was obtained from all participants or their families.

### Examinations

In the baseline survey, we collected the following information: demographic data, past medical history, history of diabetes mellitus, history of hypertension, smoking status (i.e., current or nonsmoker, with the latter comprising individuals who had never smoked and those who had ceased smoking ≤ or >3 years previously), self-reported physical activities (graded by intensity), body mass index, blood pressure, and laboratory test results, as described previously (3, 28, 29). Blood samples were obtained from most participants 3 to 7 hours after a meal and then immediately sent to our laboratory and analyzed within a day.

R-SOD activity was measured using the method of Beauchamp and Fridovich (30). The samples were prepared as described elsewhere (15). All preparation steps were carried out at 4°C. The resultant sample was subjected to the SOD activity assay with the use of a commercially available kit (SOD test Wako, Wako Chemicals Co.; currently available at Fujifilm Wako Chemicals Co.). In brief, the assay solution was composed of 0.1 mol/L phosphate buffer, pH 8.0, containing (A) 1.0 mL of 0.24 mmol/L nitroblue tetrazolium, and (B) 1.0 mL of 0.049 U/mL xanthine oxidase as a superoxide generator. After adding solutions (A) and (B) to 0.1 mL of sample, the mixture was incubated at 37°C for 20 minutes. The reaction was terminated by adding 2.0 mL of 69 mmol/L SDS solution.

The absorbance of each solution was read at 560 nm against a control with distilled water added in place of the sample. The SOD activity was calculated by subtraction of these values including with and without the sample and with and without xanthine oxidase. One unit of enzymatic activity is defined as the amount of enzyme causing 50% inhibition of the reduction of nitroblue tetrazolium to formazan observed in the blank. Samples with >85% inhibition were diluted as appropriate to avoid decreased linearity in high values of this assay method. The resultant R-SOD activities were divided by plasma hemoglobin concentration to avoid the reduced SOD activity due to decreased red blood cells and are therefore expressed as U/mg Hb.

Serum thiobarbituric acid reactive substances (TBARS), which serve as markers of oxidative stress, were also measured using the previously described method (31). Concentrations of TBARS were expressed in terms of malondialdehyde (μmol/L of serum). The substances reacting with thiobarbituric acid (TBA) in this system have been reported to be metabolites of unsaturated fatty acid hydroperoxides (32). The advantage of the present TBA assay is to isolate lipids by precipitating them with serum protein using phosphotungstic acid. By this procedure, TBA-reacting substances in serum, other than lipid peroxide, can be easily removed.

The coefficients of variation for all laboratory tests were confirmed to be <5%.

### Follow-up study

In 2004, questionnaires were mailed to the participants. Self-reports of cancer events, which included the type of cancer and the date of the diagnosis, were validated by reference to hospital records and death certificates. Most of the information was obtained during 2004 to 2005. We observed 19,634 person-years in this cohort. The primary end point was the development of malignant disease.

### Statistical methods

We compared the baseline characteristics of the study cohort using the *t* test or Mann–Whitney test for continuous variables, and the *χ*^2^ test for categorical variables between women and men and between participants who developed cancer and those who did not. We used the Cox proportional hazards model to estimate the association between baseline R-SOD activity and a future risk of cancer. Participants were categorized into quartiles according to baseline R-SOD activities, and quartile-specific HRs for cancer development were estimated. We included age, sex, smoking habit, alcohol use, physical activity, and body mass index, all of which were reported to be potential confounders for cancer development (33), as covariates in multivariable models.

Despite having data on former smokers, accurately categorizing them into high-risk or low-risk groups remains challenging due to potential unreported reasons for stopping smoking. However, because current smoking status is primarily associated with high risk, we opted to include this as a covariate. We also included systolic blood pressure (SBP) and total cholesterol levels in multivariable model 1 and immunoreactive insulin (IRI) and non–high-density lipoprotein cholesterol (non–HDL-C) levels in multivariable model 2. These variables were significantly or nearly associated with cancer risk in either the univariate analysis or the age- and sex-adjusted analyses in the present study. We also analyzed R-SOD activity as a continuous variable in the multivariable model to estimate its importance for the risk of cancer compared with other potential risk factors.

Participants who died during the follow-up period due to causes other than cancer were censored at the time of death. Their data were included in the analysis using the Cox proportional hazards model.

We used SPSS 11.01J software for Windows (SPSS) to perform the statistical analyses. A *P* value of <0.05 was considered statistically significant.

### Data availability

The data generated in this study are not publicly available because this information cannot be disclosed publicly. However, they are available from the corresponding author on reasonable request.

## Results

During the follow-up period of 10.9 years, we documented the cases of 160 participants (100 men and 60 women) who developed cancer. There were 41 patients with stomach cancer, 24 patients with colon cancer, 22 patients with lung cancer, 14 patients with prostate cancer, 10 patients with liver cancer, eight patients with pancreatic cancer, seven patients with breast cancer, six patients with malignant lymphoma, five patients with bladder cancer, four patients with bile duct cancer, 18 patients with other types of cancer, and one patient with cancer of unknown origin.

Table 1 shows the baseline characteristics of the study cohort. Significant differences were observed in current smoking habit (*P* < 0.001), alcohol use (*P* < 0.001), and physical activities (*P* < 0.001) between women and men. IRI (*P* < 0.001), total cholesterol (*P* < 0.001), HDL-C (*P* < 0.001), non–HDL-C (*P* < 0.001), and R-SOD activity (*P* < 0.001) were significantly higher in women. In contrast, SBP (*P* = 0.022), diastolic blood pressure (*P* < 0.001), hemoglobin (*P* < 0.001), glycosylated hemoglobin A1c (*P* < 0.001), triglycerides (*P* < 0.001), and TBARS (*P* = 0.003) were significantly higher in men.

**Table 1:** Baseline characteristics of the study cohort

Characteristic^a^	Total cohort	Women	Men	*P* ^b^
Number of participants	1,921	1,121	800	
Age (years)	58.7 ± 14.7	58.5 ± 14.8	59.0 ± 14.6	0.501
Diabetes (%)	5.3	3.9	7.1	0.002
Hypertension (%)	34.0	34.8	32.9	0.382
Current smoking habit (%)	22.3	2.1	50.8	<0.001
Alcohol use^c^				<0.001
Nondrinkers (%)	47.8	67.1	20.9	NA
< Two drinks/day (%)	39.7	32.6	49.5	NA
≥ Two drinks/day (%)	12.5	0.3	29.6	NA
Physical activities				<0.001
Low (%)	36.1	37.5	34.3	NA
Moderate (%)	41.2	52.5	25.5	NA
High (%)	22.6	10.1	40.3	NA
Body mass index (kg/m^2^)	22.5 ± 3.0	22.5 ± 3.1	22.5 ± 2.9	0.840
SBP (mmHg)	134 ± 21	133 ± 22	135 ± 20	0.022
DBP (mmHg)	77 ± 12	76 ± 12	79 ± 12	<0.001
Hemoglobin (g/dL)	13.5 ± 1.5	12.8 ± 1.1	14.5 ± 1.3	<0.001
HbA1c (%)	5.1 (4.9–5.5)	5.1 (4.8–5.5)	5.2 (4.9–5.6)	<0.001
IRI (μU/mL)	31 (17–58)	33 (19–60)	26 (13–55)	<0.001
Total cholesterol (mg/dL)	199 ± 36	206 ± 36	189 ± 34	<0.001
HDL-C (mg/dL)	59 ± 15	60 ± 14	56 ± 14	<0.001
Non–HDL-C (mg/dL)	138 (114–166)	143 (119–171)	131 (107–158)	<0.001
Triglycerides (mg/dL)	90 (65–129)	86 (62–121)	97 (69–145)	<0.001
TBARS (μmol/L)	4.7 (3.9–5.8)	4.6 (3.9–5.7)	4.9 (4.0–5.9)	0.003
R-SOD activity (U/mg Hb)	8.3 ± 1.8	8.5 ± 1.9	8.0 ± 1.7	<0.001

Abbreviations: DBP, diastolic blood pressure; HbA1c, glycosylated hemoglobin A1c; NA, not applicable.

a Values are expressed as mean ± SD for normally distributed variables, which were compared with the use of *t* tests; for skewed variables, which are given as a median and an IQR in parentheses, *P* values were calculated with the use of Mann–Whitney test. *P* values for the categorical variables were estimated with the use of the χ^2^ test.

b *P* < 0.05 is considered a statistically significant difference between men and women.

c One drink contains approximately 12 g of alcohol.

Table 2 shows baseline characteristics of participants who developed cancer and those who did not (expressed as with or without cancer). Age (*P* < 0.001), male sex (*P* < 0.001), current smoking habit (*P* = 0.001), hemoglobin (*P* = 0.004), and R-SOD activity (*P* = 0.022) were significantly higher in participants who developed cancer. In contrast, total cholesterol (*P* = 0.001) and non–HDL-C (*P* = 0.005) were significantly higher in participants who did not develop cancer. We also found significant differences in alcohol use (*P* = 0.036) and physical activities (*P* = 0.028) between participants who developed cancer and those who did not.

**Table 2:** Baseline characteristics of participants with or without cancer

Characteristic^a^	Participants without cancer	Participants with cancer	*P* ^b^
Number of participants	1,761	160	
Age (years)	58.1 ± 14.9	65.9 ± 10.7	<0.001
Male sex (%)	39.8	62.5	<0.001
Diabetes (%)	5.1	6.9	0.338
Hypertension (%)	33.8	36.3	0.529
Current smoking habit (%)	21.4	33.1	0.001
Alcohol use^c^			0.036
Nondrinkers (%)	48.6	39.4	NA
Drinkers < two drinks/day (%)	39.4	43.1	NA
Drinkers ≥ two drinks/day (%)	12.0	17.5	NA
Physical activities			0.028
Low (%)	35.5	42.5	NA
Moderate (%)	42.1	31.3	NA
High (%)	22.3	26.3	NA
Body mass index (kg/m^2^)	22.5 ± 3.0	22.7 ± 3.0	0.282
SBP (mmHg)	133 ± 21	136 ± 21	0.104
DBP (mmHg)	77 ± 12	79 ± 12	0.069
Hemoglobin (g/dL)	13.4 ± 1.5	13.8 ± 1.6	0.004
HbA1c (%)	5.1 (4.9–5.5)	5.2 (4.9–5.6)	0.191
IRI (μU/mL)	30 (17–58)	37 (19–68)	0.132
Total cholesterol (mg/dL)	200 ± 36	190 ± 38	0.001
HDL-C (mg/dL)	59 ± 15	58 ± 14	0.638
Non–HDL-C (mg/dL)	139 (115–167)	129 (107–155)	0.005
Triglycerides (mg/dL)	89 (64–129)	92 (66–139)	0.349
TBARS (μmol/L)	4.7 (3.9–5.8)	4.8 (4.0–5.8)	0.690
R-SOD activity (U/mg Hb)	8.3 ± 1.8	8.6 ± 2.2	0.022

Abbreviations: DBP, diastolic blood pressure; HbA1c, glycosylated hemoglobin A1c; NA, not applicable.

a Values are expressed as mean ± SD for normally distributed variables, which were compared with the use of *t* tests; for skewed variables, which are given as a median and an IQR in parentheses, *P* values were calculated with the use of the *t* test, Mann–Whitney test, and χ^2^ test.

b *P* < 0.05 is considered a statistically significant difference between participants who developed cancer and those who did not (expressed as with and without cancer, respectively).

c One drink contains approximately 12 *g* of alcohol.

Table 3 shows univariate and age- and sex-adjusted analyses of potential risk factors for the development of cancer. In the univariate analyses, we found a significantly increased risk of cancer with increasing age [hazard ratio (HR), 1.05; 95% confidence interval (CI), 1.03–1.06; *P* < 0.001], male sex (HR, 2.47; 95% CI, 1.80–3.41; *P* < 0.001), current smoking habit (HR, 1.76; 95% CI, 1.27–2.45; *P* = 0.001), alcohol use of more than two drinks a day (HR, 1.72; 95% CI, 1.10–2.68; *P* = 0.017), and SBP (HR, 1.64; 95% CI, 1.03–2.62; *P* = 0.038). Interestingly, we also found a significantly decreased cancer risk with moderate physical activity (HR, 0.60; 95% CI, 0.42–0.86; *P* = 0.006), total cholesterol (HR, 0.56; 95% CI, 0.36–0.86; *P* = 0.009), and non–HDL-C (HR, 0.55; 95% CI, 0.35–0.85; *P* = 0.007). However, in age- and sex-adjusted analyses, only R-SOD activity (HR, 1.67; 95% CI, 1.08–2.60; *P* = 0.022) and non–HDL-C (HR, 0.59; 95% CI, 0.37–0.92; *P* = 0.019) were significantly associated with increased risk of cancer. In addition, we also found that IRI in the quartile 3 population was significantly associated with increased risk of cancer as compared with the quartile 1 population (HR, 1.72; 95% CI, 1.10–2.70; *P* = 0.017), and quartile 4 was nearly significantly associated with increased cancer risk (HR, 1.55; 95% CI, 0.99–2.41; *P* = 0.055).

**Table 3:** Univariate and age- and sex-adjusted analyses of potential risk factors for the development of cancer

Variable^a^	Univariate analysis		Age- and sex-adjusted analyses	
	HR^b^ (95% CI)	*P*	HR^b^ (95% CI)	*P*
Age	1.05 (1.03–1.06)	<0.001***	NA	NA
Male sex	2.47 (1.80–3.41)	<0.001***	NA	NA
History of diabetes	1.41 (0.76–2.59)	0.277	1.10 (0.59–2.02)	0.769
History of hypertension	1.15 (0.83–1.59)	0.389	0.83 (0.60–1.15)	0.268
Current smoking habit	1.76 (1.27–2.45)	0.001**	1.25 (0.85–1.84)	0.248
Nondrinkers	1 (reference)	NA	1 (reference)	NA
Drinkers^c^ ≤ two drinks/day	1.27 (0.90–1.79)	0.170	1.20 (0.82–1.75)	0.354
Drinkers^c^ > two drinks/day	1.72 (1.10–2.68)	0.017*	1.24 (0.74–2.10)	0.416
Low physical activity	1 (reference)	NA	1 (reference)	NA
Moderate physical activity	0.60 (0.42–0.86)	0.006**	1.02 (0.69–1.50)	0.936
High physical activity	0.94 (0.64–1.38)	0.761	0.80 (0.54–1.20)	0.287
Body mass index	1.07 (0.68–1.66)	0.780	1.19 (0.76–1.85)	0.455
SBP	1.64 (1.03–2.62)	0.038*	0.97 (0.60–1.57)	0.906
DBP	1.56 (0.96–2.53)	0.072	1.25 (0.77–2.04)	0.363
Hemoglobin	1.48 (0.98–2.25)	0.065	1.21 (0.73–2.03)	0.459
HbA1c	1.50 (0.94–2.37)	0.086	0.91 (0.57–1.45)	0.684
IRI Q3 vs. Q1	1.31 (0.84–2.04)	0.232	1.72 (1.10–2.70)	0.017*
IRI Q4 vs. Q1	1.34 (0.86–2.09)	0.196	1.55 (0.99–2.41)	0.055
Total cholesterol	0.56 (0.36–0.86)	0.009**	0.66 (0.42–1.05)	0.077
HDL-C	1.03 (0.67–1.60)	0.883	1.31 (0.85–2.04)	0.224
Non–HDL-C	0.55 (0.35–0.85)	0.007**	0.59 (0.37–0.92)	0.019*
Triglycerides	1.32 (0.84–2.06)	0.228	1.12 (0.71–1.75)	0.634
TBARS	1.08 (0.70–1.67)	0.727	0.91 (0.59–1.40)	0.659
R-SOD activity	1.40 (0.91–2.16)	0.126	1.67 (1.08–2.60)	0.022*

Abbreviations: DBP, diastolic blood pressure; HbA1c, glycosylated hemoglobin A1c; NA, not applicable.

^∗^, *P* < 0.05; ^∗∗^, *P* < 0.01; ^∗∗∗^, *P* < 0.001.

a All continuous variables were categorized into quartiles, and quartile-specific HRs were calculated with the use of the Cox proportional hazards model.

b The HRs indicate the risk of quartile 4 compared with quartile 1, unless otherwise indicated. Age was calculated as a continuous variable.

c One drink contains approximately 12 *g* of alcohol.

Because these covariates are thought to be potential confounders for the relationship between baseline R-SOD activity and a future risk of cancer, we performed multivariable analysis adjusted for these variables in addition to traditional cancer risk factors including smoking habit, alcohol use, physical activity, and body mass index as covariates (33). We also found in the univariate analysis that male sex was significantly associated with the risk of cancer (refer to Table 3), and that current smoking status was more predominant in men than that in women (refer to Table 1). Therefore, we analyzed this association in women and men separately. In addition, we also found a significant difference in R-SOD activities between women and men. Therefore, we estimated cancer risk based on sex-specific quartiles of R-SOD activities.

Table 4 presents the incidence of cancer events based on sex-specific quartiles of R-SOD activity. We found that the quartile 4 group in the male cohort exhibited a higher incidence of cancer than that of females.

**Table 4:** Incidence of cancer events by sex-specific R-SOD quartile

Cohort	Total			Women			Men		
Variable	Participant (*n*)	Events (*n*)	Incidence rate (%)	Participant (*n*)	Event (*n*)	Incidence rate (%)	Participant (*n*)	Event (*n*)	Incidence rate (%)
Quartile 1	475	35	7.4	282	12	4.3	205	15	7.3
Quartile 2	487	38	7.8	281	14	5	204	26	12.7
Quartile 3	469	38	8.1	276	16	5.8	187	23	12.3
Quartile 4	490	49	10	282	18	6.4	204	36	17.6
Total	1,921	160	8.3	1,121	60	5.4	800	100	12.5

Table 5 shows multivariable-adjusted analyses of sex-specific quartiles of R-SOD activities and future risk of cancer in the total, women, and men cohorts. In multivariable model 1, adjusting for age, (sex for the total cohort), current smoking status, alcohol use, physical activity, body mass index, SBP, and total cholesterol, we found significant associations between R-SOD activity and increased risk of cancer in the total cohort (HR, 1.63; 95% CI, 1.04–2.54; *P* = 0.034) and the men cohort (HR, 2.49; 95% CI, 1.35–4.61; *P* = 0.004), but not in the women cohort. In multivariable model 2, adjusting for age, (sex for total cohort), current smoking status, alcohol use, physical activity, body mass index, IRI, and non–HDL-C, this association did not change appreciably (HR, 1.61; 95% CI, 1.03–2.52; *P* = 0.037 for the total cohort; and HR, 2.49; 95% CI, 1.35–4.59; *P* = 0.003 for the men cohort). We also did not find any association between R-SOD activity and cancer risk in women in this analysis.

**Table 5:** Multivariable-adjusted analyses of sex-specific quartiles of R-SOD activities and cancer risk

Cohort	Comparison	Multivariable model 1^a^		Multivariable model 2^b^	
		HR^c^ (95% CI)	*P*	HR^c^ (95% CI)	*P*
Total^d^	Q2 vs. Q1	1.10 (0.70–1.76)	0.661	1.10 (0.69–1.75)	0.686
	Q3 vs. Q1	1.21 (0.76–1.92)	0.423	1.20 (0.75–1.91)	0.444
	Q4 vs. Q1	1.63 (1.04–2.54)	0.034	1.61 (1.03–2.52)	0.037
Women	Q2 vs. Q1	1.24 (0.57–2.71)	0.583	1.23 (0.57–2.69)	0.597
	Q3 vs. Q1	1.33 (0.63–2.83)	0.458	1.33 (0.63–2.83)	0.457
	Q4 vs. Q1	1.48 (0.71–3.10)	0.299	1.46 (0.70–3.05)	0.320
Men	Q2 vs. Q1	1.67 (0.87–3.20)	0.122	1.68 (0.88–3.21)	0.119
	Q3 vs. Q1	1.62 (0.84–3.12)	0.149	1.63 (0.85–3.15)	0.142
	Q4 vs. Q1	2.49 (1.35–4.61)	0.004	2.49 (1.35–4.59)	0.003

a Multivariable model 1 includes age, current smoking habit, alcohol use, physical activities, body mass index, SBP, and serum total cholesterol as covariates.

b Multivariable model 2 includes age, current smoking habit, alcohol use, physical activities, body mass index, plasma IRI, and non–HDL-C as covariates.

c Baseline R-SOD activities in the total cohort, women, and men populations were separately divided into quartiles based on sex, and HRs of each quartile population were estimated with the use of the Cox proportional hazards model using quartile 1 as a reference group.

d For the total cohort, sex was also used as a covariate in addition to the other variables.

Our study is an observational study. Therefore, we should consider the possibility of underlying cancers that may have caused increased R-SOD activities at the baseline survey. To reduce the potential bias from the effects of preexisting illness on R-SOD activities, we performed a sensitivity analysis.

Table 6 shows multivariable-adjusted analyses of sex-specific quartiles of R-SOD activities and future risk of cancer after excluding cancer events within 5 years of the baseline survey; multivariable-adjusted HRs of sex-specific baseline R-SOD activity quartile 4 are compared with quartile 1 for future risk of cancer in the total, women, and men cohorts. In this analysis, we excluded 57 participants with cancer events during the 5 years after the baseline survey. After adjusting for age (and sex for the total cohort), current smoking status, alcohol use, physical activity, body mass index, IRI, and non–HDL-C, we observed significantly stronger associations between R-SOD activities and cancer risk in the male cohort compared with the results of the analysis that included cancer events within the initial 5-year period. Therefore, increased baseline R-SOD activities seem to be useful in predicting cancer risk beyond 5 years from the baseline survey.

**Table 6:** Multivariable-adjusted analyses of sex-specific quartiles of R-SOD activities and cancer risk, after excluding the events within 5 years from the baseline survey

Cohort	Comparison	Multivariable model 1^a^		Multivariable model 2^b^	
		HR^c^ (95% CI)	*P*	HR^c^ (95% CI)	*P*
Total^d^	Q2 vs. Q1	1.10 (0.70–1.76)	0.661	1.34 (0.75–2.40)	0.328
	Q3 vs. Q1	1.21 (0.76–1.92)	0.423	1.42 (0.78–2.58)	0.246
	Q4 vs. Q1	1.78 (1.00–3.18)	0.052	1.78 (1.00–3.17)	0.052
Women	Q2 vs. Q1	1.67 (0.69–4.07)	0.257	1.67 (0.69–4.05)	0.260
	Q3 vs. Q1	1.17 (0.46–2.99)	0.741	1.17 (0.46–2.98)	0.747
	Q4 vs. Q1	1.03 (0.39–2.70)	0.956	1.01 (0.39–2.65)	0.983
Men	Q2 vs. Q1	2.68 (0.87–3.20)	0.042	2.74 (1.06–7.08)	0.037
	Q3 vs. Q1	2.76 (0.84–3.12)	0.038	2.87 (1.10–7.46)	0.031
	Q4 vs. Q1	4.56 (1.84–11.26)	0.001	4.64 (1.88–11.45)	0.001

a Multivariable model 1 includes age, current smoking habit, alcohol use, physical activities, body mass index, SBP, and serum total cholesterol as covariates.

b Multivariable model 2 includes age, current smoking habit, alcohol use, physical activities, body mass index, plasma IRI, and non–HDL-C as covariates.

c Baseline R-SOD activities in the total cohort, women, and men populations were separately divided into quartiles based on sex, and HRs of each quartile population were estimated with the use of the Cox proportional hazards model using quartile 1 as a reference group.

d For the total cohort, sex was also used as a covariate in addition to the other variables.

Table 7 shows multivariable-adjusted HRs, including R-SOD as a continuous variable, for future risk of cancer in the total, women, and men cohorts. Body mass index (HR, 1.08; 95% CI, 1.02–1.14; *P* = 0.011 for the total cohort; and HR, 1.13; 95% CI, 1.04–1.21; *P* = 0.002 for the men cohort) and R-SOD activity (HR, 1.14; 95% CI, 1.06–1.22; *P* = 0.001 for the total cohort; and HR, 1.15; 95% CI, 1.06–1.26; *P* = 0.002 for the men cohort) were significantly associated with increased risk of cancer. In contrast, non–HDL-C was associated with a decreased risk of cancer in the total cohort (HR, 0.99; 95% CI, 0.99–1.00; *P* = 0.004) and the men cohort (HR, 0.99; 95% CI, 0.98–1.00; *P* = 0.001). Therefore, among various cancer risk factors, male sex, increased body mass index, decreased non–HDL-C, and increased R-SOD activity were found to be major concerns for cancer development in this population.

**Table 7:** Multivariable-adjusted HRs including R-SOD activity as a continuous variable for future risk of cancer in the total, women, and men cohorts

Variable^a^	Total cohort		Women cohort		Men cohort	
	HR (95% CI)	*P*	HR (95% CI)	*P*	HR (95% CI)	*P*
Age	1.05 (1.04–1.07)	<0.001***	1.05 (1.02–1.07)	<0.001***	1.06 (1.04–1.08)	<0.001***
Male sex	2.31 (1.49–3.58)	<0.001***	NA		NA	
Current smoking habit	1.30 (0.88–1.91)	0.186	ND	0.970	1.50 (0.99–2.28)	0.055
Nondrinkers^b^	1 (reference)	0.622^c^	1 (reference)	0.583^c^	1 (reference)	0.734^c^
Drinkers (≤ two drinks/day)	1.19 (0.81–1.75)	0.373	1.35 (0.77–2.38)	0.299	1.13 (0.67–1.90)	0.658
Drinkers (> two drinks/day)	1.04 (0.61–1.77)	0.884	ND	0.992	0.94 (0.51–1.71)	0.828
Low physical activity^b^	1 (reference)	0.289^c^	1 (reference)	0.637^c^	1 (reference)	0.366^c^
Moderate physical activity	1.02 (0.69–1.52)	0.905	1.14 (0.65–2.00)	0.643	0.88 (0.50–1.55)	0.652
High physical activity	0.75 (0.50–1.12)	0.161	0.73 (0.28–1.92)	0.522	0.72 (0.45–1.14)	0.159
Body mass index	1.08 (1.02–1.14)	0.011*	1.02 (0.93–1.12)	0.634	1.13 (1.04–1.21)	0.002**
IRI	1.00 (0.98–1.02)	0.912	0.99 (0.95–1.04)	0.807	1.00 (0.98–1.03)	0.773
Non–HDL-C	0.99 (0.99–1.00)	0.004**	1.00 (0.99–1.01)	0.602	0.99 (0.98–1.00)	0.001**
R-SOD activity	1.14 (1.06–1.22)	0.001**	1.10 (0.98–1.24)	0.118	1.15 (1.06–1.26)	0.002**

Abbreviations: NA, not applicable; ND, not determined.

^∗^, *P* < 0.05; ^∗∗^, *P* < 0.01; ^∗∗∗^, *P* < 0.001.

a All variables were included in the Cox proportional hazards model to estimate the risk associated with each variable in the model.

b HRs and *P* values were calculated with the use of nondrinkers for alcohol use and low physical activity for physical activities as reference groups.

c Expressed as *P* values among three categories of drinkers and those of physical activities.

## Discussion

In this community-based cohort study, we found that increased R-SOD activity was associated with a future risk of cancer, even after adjustment for potential cancer risk factors. This association was statistically significant in men but not in women. We also found a similar result in the analysis that excluded participants who developed cancer within 5 years of the baseline survey, suggesting that this association was unaffected by the effects on R-SOD activities of cancer present at the time of the baseline survey.

ROS promote both cell survival and apoptosis. At high concentrations, ROS cause DNA damage, whereas at low concentrations, they act as a signal transduction molecule, leading to antiapoptotic signals (34). SOD catalyzes superoxide anion to produce hydrogen peroxide, which acts as a signal transduction molecule and upregulates VEGF expression and angiogenesis in cancer cells (35). Therefore, SOD seems to contribute to cell proliferation through generation of hydrogen peroxide. Furthermore, in clinical settings, patients with colon cancer with an increased level of SOD activity in the cancer tissue were found to have a poor postoperative prognosis (26), and secondary induction of SOD in tumors *in vivo* can be associated with an aggressive malignant transformation likely due to the altered redox status of the malignant cells (36). These findings indicate that induction of antioxidative enzymes such as SOD in cancer cells seems to favor their survival. Thus, the antioxidative enzyme that protects normal cells may also protect cancer cells against oxidative stress.

Owing to their detrimental effects on cells, ROS have long been believed to contribute to cancer cell development (4). However, recent clinical studies have shown that antioxidant vitamins (e.g., vitamin E, β-carotene, and selenium), which can eliminate ROS, have no effect on reducing incidence of cancer; instead, they increased the risk of cancer in men (37, 38). In the present study, we found increased R-SOD activities to be associated with increased risk of cancer in men but not in women. Compared with women, men had higher levels of TBARS and lower R-SOD activities, indicating increased oxidative stress in men. Therefore, our results suggest that increased antioxidative enzymes such as R-SOD may contribute to cancer development, which would be consistent with the findings of the clinical studies (37, 38).

Experimental studies have also shown that increased ROS levels are predominantly observed in males rather than females (39–41). Furthermore, a recent study has shown increased ROS production and decreased SOD activity in the male umbilical vein compared with the female umbilical vein, in response to TNFα (41, 42). These findings support the idea that ROS and antioxidant systems differ substantially between men and women. In the present study, we found that an enhanced antioxidant system, specifically SOD activity, is associated with an increased risk of cancer in the general population. In contrast, no significant relationships were found between metabolites of oxidized lipids, such as TBARS, and cancer risk.

The relationship between increased R-SOD activity and future risk of cancer was more pronounced in the male cohort when those who had cancer events within the first 5 years of the baseline survey were excluded. This finding suggests that increased R-SOD activity may indicate an increased risk of cancer independent of the effects of preexisting cancer.

### Limitations

Our study has several limitations. First, cancer cells may regulate their redox potential to favor their survival, which is determined not only by the amount of SOD but also by the amounts of superoxide anion, catalase, and glutathione peroxidase. However, we did not measure these redox-sensitive markers.

Second, we measured SOD activity in normal cells, but not in cancer cells. Nevertheless, it is noteworthy that the activity of the antioxidative enzyme in red blood cells, which are derived from stem cells, could be related to a future risk of cancer, suggesting that systemic redox status may be critical for cancer development and survival. The multivariable-adjusted analysis, which excluded participants who developed cancer within the first 5 years of follow-up, may support this idea.

Third, we did not collect any information on diet or supplements from the participants, which are important factors for the development of cancer. Therefore, we were not able to include dietary factors as covariates in multivariable models in this study.

Fourth, we found a sex-based difference in cancer risk in this population. However, the population includes a limited number of female smokers, which could have contributed to the reduced incidence of cancer events among women. Furthermore, we included former smokers in the nonsmoker group due to insufficient data with regard to their smoking history, including duration of smoking habit and reasons for stopping smoking. Therefore, the stratification of participants in the present study based on current smoking status versus nonsmoking status may not fully account for the effects of smoking as a confounding factor.

Fifth, we used serum TBARS as an oxidative stress marker in the present study. However, using a DNA damage marker such as urinary 8-hydroxy-2′-deoxyguanosine might have been more favorable, as it is reported to be associated with cancer development (43, 44). Unfortunately, at the time of the baseline survey, no assay kit for 8–hydroxy-2′-deoxyguanosine was available for the large number of cohort samples.

Finally, for the collection of data on cancer events, we relied on self-reported information obtained from questionnaires, which could potentially result in under-reporting. We were able to validate the events by reference to hospital records and death certificates; however, we were unable to detect cancer events in patients who did not report them.

### Conclusion

Increased R-SOD activities were associated with a future risk of cancer in a general Japanese population, suggesting that systemic redox status may be related to cancer development. R-SOD activities may be useful for predicting cancer risk, particularly in the male population.
